# Significant Fatigue Life Enhancement in Multiscale Doubly-Modified Fiber/Epoxy Nanocomposites with Graphene Nanoplatelets and Reduced-Graphene Oxide

**DOI:** 10.3390/polym12092135

**Published:** 2020-09-18

**Authors:** Mohammad Rafiee, Somayeh Hosseini Rad, Fred Nitzsche, Jeremy Laliberte, Michel R. Labrosse

**Affiliations:** 1 Department of Mechanical Engineering, University of Ottawa, Ottawa, ON K1N 6N5, Canada; Michel.labrosse@uottawa.ca; 2 Department of Mechanical Engineering, Polytechnique Montreal, Montreal, QC H3T 1J4, Canada; somayeh.hosseini-rad@polymtl.ca; 3 Department of Chemical Engineering, Polytechnique Montreal, Montreal, QC H3T 1J4, Canada; 4 Department of Mechanical and Aerospace Engineering, Carleton University, Ottawa, ON K1S 5B6, Canada; fred.nitzsche@carleton.ca (F.N.); Jeremy.laliberte@carleton.ca (J.L.)

**Keywords:** low cycle fatigue, reduced-graphene oxide, graphene nanoplatelets, nanoparticle, composite laminates

## Abstract

We report the fatigue behavior of a novel multiscale fiberglass/epoxy composite modified with reduced-graphene oxide (rGO) and graphene nanoplatelets (GNP). A novel and cost-effective fabrication method based on vacuum assisted resin transfer molding (VARTM) method was used for manufacturing the composite laminates. Morphological and mechanical analysis of composites showed a successful dispersion of nano-fillers and a remarkable improvement in fatigue life of the nanocomposites. The experimental results revealed that all rGO concentrations resulted in a significant increase in fatigue life of the nanocomposites. These enhancements can be explained by the creation of stronger links between the nanoparticles fiberglass and epoxy. The experimental results also showed that lower concentrations of GNPs lead to an increase in fatigue life of nanocomposites; however, a decrease in their fatigue life can be seen at higher loadings.

## 1. Introduction

Reinforced epoxy polymers are one of the most favorable light-weight materials with several engineering applications including the aerospace and automotive industries [1]. In the case of light-weight structures, specific mechanical properties are more important, therefore investigating the strength of the reinforced epoxy polymers under different operating conditions and types of loading has been a subject of several recent studies. Fatigue failure, as one of the most common reasons for break down, has been investigated as a vital property in designing the epoxy composites subjected to cyclic loads.

Graphene-based nanomaterials such as reduced-graphene oxide (rGO) and graphene nanoplatelets (GNPs) have excellent thermo-electro-mechanical properties and their addition to the polymer matrix potentially provides improved mechanical, thermal and electrical properties. GNPs were observed as a reinforcement agent and a promising candidate for replacing carbon nanotubes (CNTs) regarding cost and final properties of nanocomposites [2,3,4,5]. Properties of GNPs can be affected by their synthesis methods.

Several researchers have studied fatigue life of epoxy nanocomposites which mostly incorporate CNTs (e.g., [6,7,8,9,10,11,12,13,14,15]) and rarely rGO [16] and GNPs [14,16,17,18,19,20,21,22,23]. Rafael et al. [24] appeared to be the first researchers studying the effect of graphene nanoparticles on fatigue and fracture properties of two-phase epoxy polymers reinforced with different weight percentages of GNPs. The authors showed that 0.125 wt.% of modified graphene sheets was shown to improve the fatigue life of epoxy nanocomposite. Bortz et al. [16] investigated the fatigue life of GO/epoxy composites and by incorporating a small percentage of graphene oxide (≤1 wt.%), they increased the fatigue life of nanocomposite by as much as 1580% over the baseline composition. Flexural fatigue properties of epoxy-based nanocomposites containing a combination of two types of nanomaterials, synthesized GNPs and carbon nanofibers, have been studied by Shokrieh et al. [21]. In their study, incorporation of hybrid nanoparticles showed a significant enhancement of fatigue properties for the composites compared to results achieved by incorporating GNPs or CNF separately into the matrix. In another study, Shokrieh et al. [20] investigated the mechanical properties of GNPs/epoxy nanocomposites under flexural fatigue bending tests. In the authors’ work, a remarkable improvement in fatigue property of the nanocomposites was reported. For example, epoxy resin specimens with 0.25 wt.% of graphene and fatigued at a stress ratio of 0.43 revealed a 27.4-fold improvement in fatigue life in comparison with the neat epoxy resin. Ladani et al. [22] investigated the effects of the shape and concentration of nanoscale carbon fillers such as GNPs and CNFs on the fracture energy and crack propagation in fatigue testing of epoxy nanocomposites. The numerical results in their study confirmed the effectiveness of CNFs or GNPs nanofillers as a reinforcement agent in enhancing the resistance of the matrix to the phenomenon of crack growth during fatigue. They also showed that at higher energy release-rates, the CNFs played a larger role at resisting the fast fatigue crack growth when compared to the GNPs. As a mechanism of improving the fatigue property of epoxy nanocomposites, Bhasin et al. [23] assessed the effectiveness of electric field orientation of GNPs. The authors showed that the orientation of the GNPs caused by an electric field led to more enhancement in the fatigue crack growth resistance of the resin compared to that produced by adding randomly-aligned GNPs, especially in the near threshold region.

Despite the importance of multiscale composite laminates, few studies have focused on their fatigue life assessment when reinforced with graphene nanomaterials [14,17,18]. The current manufacturing method reported to produce such composites are often expensive and not scalable to large productions. For instance, the preparation and fatigue properties of fiberglass/epoxy composites with different loading amounts of GNPs were studied by Yavari et al. [17]. Only ~0.2 wt.% (regarding the epoxy resin) of GNP improved the fatigue property in the mode of flexural bending by up to 1200-fold. Contrarily, under uniaxial mode of the test, the GNP additives led to ~3–5-fold enhancement. Shen et al. added GNPs to epoxy composites and carbon fiber/epoxy composite laminates to improve their fatigue lives [18]. Comparing neat laminates with those incorporating 0.25 wt% of GNPs showed that even adding a small amount of this additive led to an increase in fatigue property at all cyclic-stress levels of the fatigue test. Fatigue behavior of carbon fiber reinforced epoxy after incorporation of multiwalled carbon nanotubes and GNPs as well as damage mechanisms were investigated by Knoll et al. [14]. They studied the effect of nanoparticles in the high cycle regime and found that the addition of low amounts of the nanoparticles resulted in a significant increase of fatigue life for all load levels.

In light of the remarkable characteristics of graphene materials, the addition of such nanoparticles into the matrix of polymeric composites could be considered an effective way to improve the fatigue life of multiscale composites. To the best knowledge of authors, the study of the fatigue lifetime of multiscale composites is limited to a few studies on GNPs; the influence of rGO on fatigue properties of multiscale composite laminates has not been studied experimentally. In addition, the manufacturing technology presented herein is low-cost and scalable.

This study is an extension of the authors’ previous work [25] and investigates the fatigue life of nanocomposites when incorporating rGO and GNP nanomaterials. A new manufacturing approach for preparation of nanocomposites was applied which included sizing glass-fiber fabrics as well as spraying nanoparticle into the resin. The low-cost and scalable vacuum assisted resin transfer molding (VARTM) method was used for manufacturing laminates. Several panels were fabricated using this technology. The effect of added nanomaterials and their percentage on fatigue life of three-phase multi-scale glass-fiber reinforced polymers (GFRPs) reinforced with rGO and GNP were investigated.

## 2. Materials and Methods

### 2.1. Materials

Graphene oxide was purchased from Ablalonyx, Norway and through thermal reduction, rGO was produced. Neat GNPs with the mean thickness of 1.5 nm and diameter of 5 µm were provided from Cheap Tubes Inc. (Cambridgeport, VT, USA). Unidirectional E-Glass fabrics were purchased from NMG (Wanli Village, China). The diethyltoluenediamine (DETDA) curing agent PT-2712 Part B1 and diglycidyl ether of bisphenol-A (DGEBA) epoxy resin PT-2712 Part A was provided by PTM&W Industries, Inc., Santa Fe Springs, CA, USA. As per manufacturer recommendation, mixing weight ratio of epoxy resin Parts A and B was 100:22.

### 2.2. Preparation of Multi-Scale Nanocomposites

#### 2.2.1. Two-Phase Graphene/Epoxy Composites

The GFRP nanocomposites reinforced with GNPs and rGO were prepared with different loading percentage of nanoparticles as described in Table 1. Nanoparticles were added into the composites in two steps, first for sizing the fabrics and second for reinforcement of the matrix. Table 2 shows the loading of nanoparticles for each step. To allow an efficient dispersion of nanoparticles, acetone was chosen as it is reported to have a satisfactory result due to its proper boiling point [1] and no negative effect on the pre-curing process [26].

Dispersion of nanoparticles in acetone (1 g/L) was performed by sonication using an ultrasonic probe (Q700 Sonicator, Qsonica LLC, Newtown, CT, USA) for 90 min, 20 s ON pulses followed by 10 s OFF cycles for controlling the temperature. The sizing of the fibers using a spray gun with the desired amount of mixture (14 g/ft2 of both sides of fabric) was performed inside a fume hood, following 24 h rest to ensure full evaporation of the solvent.

The rest of the solution was stirred at 400 rpm for 12 h to achieve a better dispersion state. For evaporation of the solvent, the mixture was mixed with a magnetic stirrer for 4 h at 70 °C followed by drying in a vacuum chamber for 30 min to complete the solvent elimination. To control premature curing, the resin and curing agents were mixed at room temperature with a hardener (low viscosity DETDA) to epoxy ratio of 22:100. Using a mechanical stirrer, the solution was mixed at 800 rpm for 30 min and degassed under vacuum at room temperature for about 30 min. The final solution was then used as an infusion resin for the fabrication of the nanocomposites.

#### 2.2.2. Three-Phase Laminated Composites

The composites were fabricated with the nanomaterial-modified resin through the VARTM method. Fiberglass fabrics (three layers) in cross ply form (0°,90°,0°) were used where 0° was the main direction of testing. Curing of infused nanomaterial/epoxy composites occurred at ambient temperature for 15 h followed by a post cure rate of 1 °C/min, held every 20 °C for an hour. The final step was resting samples at 100 °C for three hours to eliminate any possible thermal history.

Samples were then cut into the desired size (1.9 ± 0.1 mm in thickness) by waterjet. Fiber volume and weight fraction percentages were calculated according to ASTM D3171 and found to be 50% and 70%, respectively. The material properties of each material used in the fabrication of the multi-scale composites are given in [27]. More details regarding the process flow and material structure of the developed composites are also given in [27].

## 3. Characterization

### 3.1. Morphological Study

The dispersion state of carbon nanomaterials and analysis of fracture surfaces are characterized using a TECNAI Spirit transmission electron microscope (TEM, FEI Company, Hillsboro, OR, USA) and a Zeiss EVO10 scanning electron microscope (SEM, Carl Zeiss AG, Oberkochen, Germany), respectively.

### 3.2. Tensile Properties

All tensile tests were performed on a universal testing machine (Instron 4482, Instron, Canton, OH, USA) according to ASTM D3039. For increasing the accuracy of the test, an Advanced Video Extensometer (Instron, Canton, OH, USA) was used. The specimen geometry had a rectangular cross-section of 25 mm wide by 3 mm ± 0.1 thickness and a test gauge length of 150 mm.

### 3.3. Fatigue

According to ASTM D3479, five specimens of 150 × 25 × 3 mm^3^ were prepared for the fatigue test. The uniaxial tension–tension loading low cyclic fatigue tests were performed on an Instron 8801 machine (Instron, Canton, OH, USA). The sinusoidal load cycle was with a frequency of 4Hz with a constant stress ratio (*R*) of 0.1. The stress ratio *R* is defined as *R* = σ_min_ = σ_max_, where σ_max_ and σ_min_ are the highest and lowest applied stresses, respectively. For all the tests, the fatigue load was kept up to the failure.

## 4. Results and Discussion

**Figure 1:**
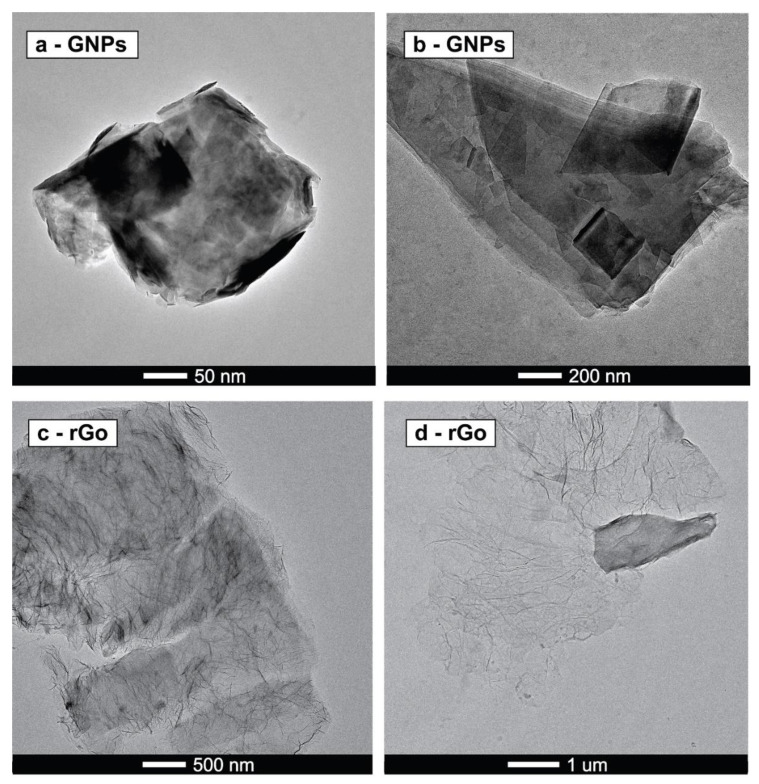
TEM graphs of nanoparticles: (**a**,**b**) GNPs and (**c**,**d**) rGO.

By using the TEM technique, localization of nanoparticles in whole samples was observed, showing proof of interaction due to van der Waals forces (Figure 1).

**Figure 2:**
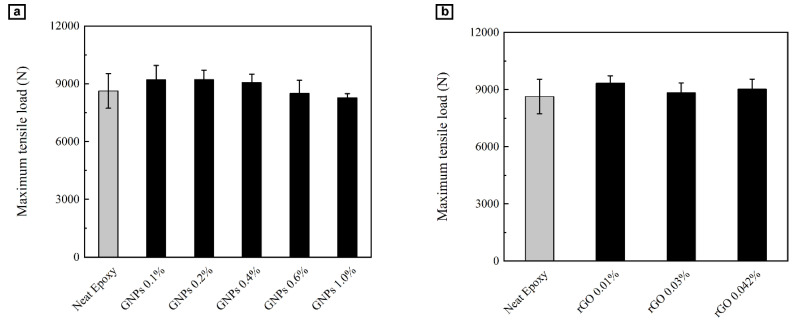
Maxiumum tensile load capacity of nanocomposite beams containing (**a**) graphene nanoparticles (GNP) and (**b**) reduced-graphene oxide (rGO).

Tensile test was used to evaluate the mechanical properties of graphene-reinforced glass fiber/epoxy composites. As seen in Figure 2, maximum tensile load of glass fiber/neat epoxy with incorporation of graphene nanoparticles increases from 8.63 ± 0.9 kN to higher levels of 9.22 ± 0.48 kN in the case of 0.1 wt.% of graphene nanoplatelets concentration, representing an improvement of 6.8%. In a similar way, specimens with 0.01 wt.% of rGO exhibited an 8.2% enhancement in final load capacity. These results confirm enhanced ultimate tensile load capacities of nanocomposites.

**Figure 3:**
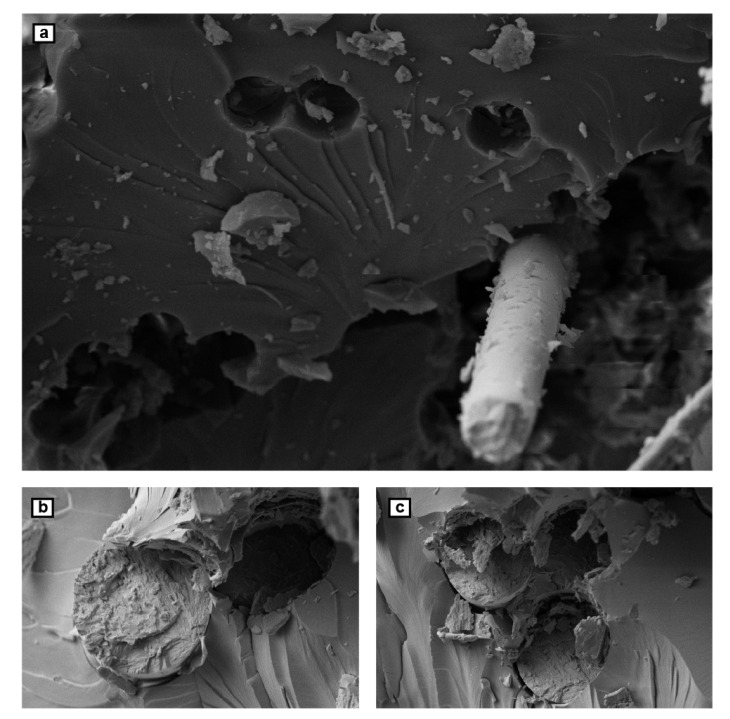
SEM images of the fractured surface of the tensile-tested samples: (**a**) the failure surface of unmodified epoxy composites; (**b**,**c**) the surface of the developed nanocomposites.

Fracture surface of the tensile-tested samples was investigated by SEM to define the morphology and interfacial strength between different phases of the nanocomposite. Figure 3 shows micrographs of pulled out fibers and their previous places inside the matrix which are similar to those observed in tensile failed samples [27]. In Figure 3a, fracture surface of glass fiber/epoxy composite is shown as a reference indicator. On the surface of these fibers, few traces of the epoxy can be observed, however the holes left by the pulled-out fibers appear as fine circles. These two observations suggest a non-uniform stress distribution in nanocomposite during tensile load and lack of load transfer between reinforcement and matrix phases.

In contrary, Figure 3b shows a better adhesion strength between different phases in cases of nanocomposites. There are no gaps or holes showing adhesion failure between the fibers and the matrix. The graphene-modified glass fibers and the epoxy resin consisting of nanoparticle additives acted as a single component during breakage which suggests accordingly strong bonding. Non-symmetric shapes of the holes and the rough surface of the fibers indicates a cohesive structure of nanocomposites, offering a proper load transfer between the epoxy matrix and reinforcement phase which can adsorb more energy before failure of the sample. This improvement can be explained by the anchoring effect of nanoparticles existing in the interface of the two phases.

**Figure 4:**
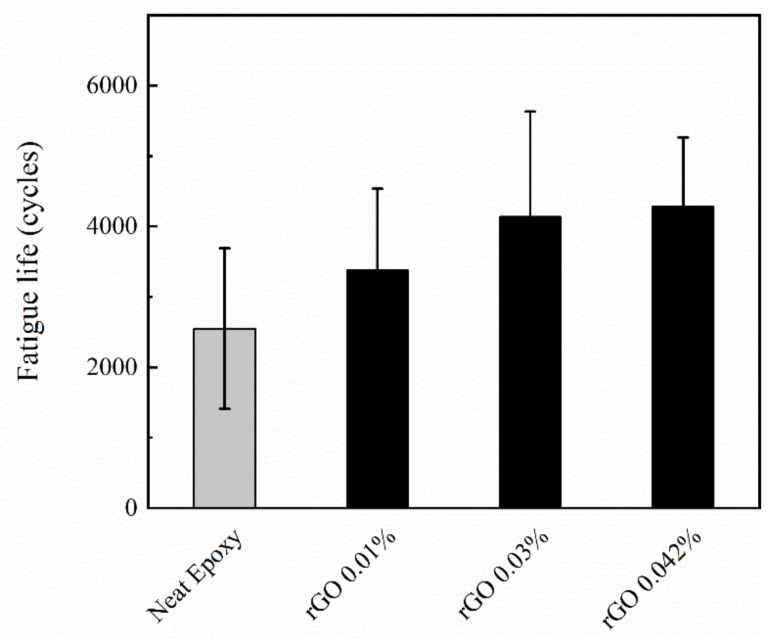
Effect of reduced-graphene oxide loading on fatigue life of multiscale GFRP nanocomposites.

**Figure 5:**
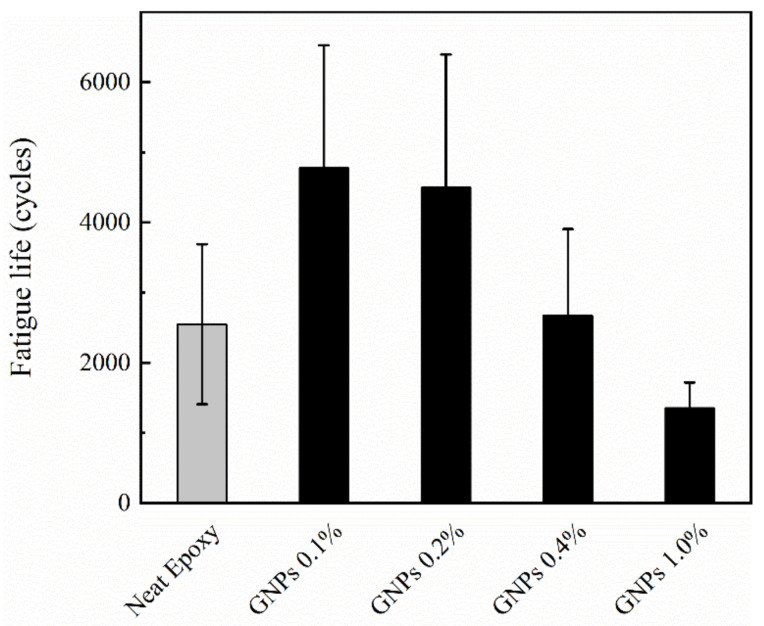
Influence of graphene nanoplatelets concentration on fatigue life of multiscale GFRP nanocomposites.

To study the effect of carbon nanomaterials on the low-cycle fatigue behavior of glass fiber/epoxy nanocomposites, tension–tension cycling test was used. The data from the fatigue tests are illustrated in Figure 4 and Figure 5. The measured fatigue life of the developed composites is also summarized in Table 3.

The influence of rGO loading on fatigue life of multiscale GFRP nanocomposites is shown in Figure 4. As depicted in this graph, all of the rGO loadings resulted in significant enhancement in fatigue properties of the nanocomposites. For instance, an increase of 68% was observed for the loading of 0.042 wt.% of rGO. These enhancements can be related to the formation of more links between the nanoparticles and the GFRP. Accordingly, better bonding led to stronger resistance to failure and enhancement of the final properties of the composites. This trend was also observed in References [16,28].

The improvement to the fatigue resistance of the multiscale nanocomposites was also due to other toughening mechanisms induced by the carbon nanofillers which were operative under cyclic loading. Similar to the case of tensile loading described above, a combination of intrinsic and extrinsic toughening mechanisms was operative under cyclic loading. These mechanisms were (a) debonding of the nanofillers, (b) plastic void growth initiated by the debonded nanofillers in the process zone ahead of the crack tip, (c) crack bridging and pull-out of the nanofillers behind the crack tip and (d) rupture of the nanofillers behind the crack tip.

The influence of GNP loading on the fatigue life of multiscale GFRP nanocomposites was also investigated and is shown in Figure 5. At lower concentrations of nanoparticles, an improvement in fatigue property of nanocomposite was observed; however, a decrease in fatigue life can be seen at higher loading. This may be due to agglomeration of nanoparticles at higher loadings which results in anisotropic material properties and crack initiation.

The measured fatigue life of the composites was compared to that of the neat epoxy and reported as percent relative difference (% Relative Difference) in Table 3. The average values listed in Table 3 were calculated from five specimens. A One-way Analysis of Variance (ANOVA) and subsequent post-hoc Tukey comparisons were performed using SPSS Statistics software in order to evaluate the effect of the composition of the composites (GNP and rGO content) on the load capacity of the developed samples (Table 3). An alpha level (significance level) of 0.05 was used for all statistical tests. The *p*-values obtained from post-hoc Tukey comparisons are listed in Table 3. Each *p*-value represents the results of the comparison of the properties between neat epoxy and the corresponding composition. *P*-values were compared with the alpha level to determine whether or not the observed differences were statistically significant. If the *p*-value is less than or equal to the alpha (*p* ≤ 0.05), then the observed differences are considered as statistically significant. ANOVA results showed that there was a statistically significant effect of the composition of the nanocomposites between lowest (GNP 0.1 wt.%) and highest (GNP 1.0 wt.%) percentage of GNPs. However, there was no statistically significant difference between fatigue life of the nanocomposite samples with respect to the neat epoxy. Due to the nature of low cycle fatigue that leads to high standard deviation, the statistical significance of difference becomes lower in this analysis.

## 5. Conclusions

Two different types of nanofillers, rGO and GNP were used as reinforcement agents in fiber/glass epoxy composites and their fatigue lives were investigated. Multiscale laminated fiberglass/epoxy composites were prepaid by VARTM method. Additional reinforcement of the matrix was applied by a novel approach of spraying resin with nanoparticles on the glass fiber before preparing composites. Morphological and mechanical analysis of composites showed a successful dispersion of nanofillers and a remarkable improvement in fatigue life of the nanocomposites. A dramatic increase of 68% was obtained for the loading of 0.042 wt.% of rGO. The source of this improvement can be raised from better adhesion and forming several linkages between nanoparticles and the GFRP, leading to a stronger load transfer between the reinforcement agents inside the matrix and nanocomposite that is more resistant to failure. While lower percentage of the GNPs showed significant increases in fatigue life of nanocomposites, higher percentages resulted in less improvements. Lower surface area due to agglomeration of GNPs at higher loadings may be the reason behind this observation.

## Figures and Tables

**Table 1:** Concentration of additives used for reinforcement of composites.

Reinforcement	wt% of DGEBA	vol.% of DGEBA	wt% of Matrix (DGEBA+ DETDA)	vol.% of Matrix (DGEBA+ DETDA)	wt% of Total (DGEBA+ DETDA+ Fibers)	vol.% of Total (DGEBA+ DETDA+fibers)
GNP	0.1	0.55	0.08	0.44	0.02	0.22
	0.2	1.1	0.16	0.88	0.05	0.44
	0.4	2.2	0.33	1.76	0.1	0.88
	1	5.5	0.82	4.4	0.25	2.2
rGO	0.01	1.1	0.008	0.88	0.002	0.44
	0.03	3.3	0.025	2.64	0.007	1.32
	0.042	4.6	0.034	3.7	0.010	1.85

**Table 2:** Amount of nanoparticle used for sizing and infusion of fiberglass/epoxy composites.

Reinforcement	wt.% of DGEBA	vol.% of DGEBA	Sprayed Vol. %	Infusion Vol.%
GNP	0.1	0.55	0.275	0.275
	0.2	1.1	0.55	0.55
	0.4	2.2	1.1	1.1
	1	5.5	4.4	1.1
rGO	0.01	1.1	0.55	0.55
	0.03	3.3	2.2	1.1
	0.042	4.6	3.5	1.1

**Table 3:** Load capacity of the developed samples and results of Tukey post-hoc comparisons (*p*-value) ^a^.

Composition	Low Cycle Fatigue Life (Cycles)	% Relative Difference	*p*
Neat Epoxy	2549 ± 1141		
GNPs 0.1%	4779 ± 1750	2229	0.297
GNPs 0.2%	4496 ± 1899	1947	0.394
GNPs 0.4%	2413 ± 1275	−136	1.000
GNPs 1.0%	1685 ± 940	−865	0.942
rGO 0.01%	2706 ± 1376	156	0.999
rGO 0.03%	3740 ± 1654	1191	0.638
rGO 0.042%	4283 ± 981	1734	0.458

^a^*p*-value stands for the comparison between the properties of neat epoxy and each nanocomposite composition.
